# Antimicrobial potential of *Actinomycetes *by NRPS and PKS-I pathways

**DOI:** 10.1186/1753-6561-8-S4-P175

**Published:** 2014-10-01

**Authors:** Maria Lucila Hernández- Macedo, Erick Nunes Barreto, Ana Carolina de Souza Cavalcanti, Rafael Salomão da Silva, Erika Cristina Teixeira dos Anjos Brandão, Roberta Pereira Miranda Fernandes, Viviane Talamini, Leandro Eugenio Cardamone Diniz, Marcelo Ferreira Fernandes

**Affiliations:** 1 Programa de Pós-Graduação em Biotecnologia Industrial, Universidade Tiradentes/Instituto de Tecnologia e Pesquisa, Aracaju, SE, 49032-490, Brazil; 2 Embrapa, Tabuleiros Costeiros, Aracaju, SE, 49025-040, Brazil; 3 Programa de Pós-Graduação em Biotecnologia de Recursos Naturais, Universidade Federal de Sergipe, São Cristóvão, SE, 49100-000, Brazil; 4 Embrapa, Tabuleiros Costeiros, Aracaju, SE, 49025-040, Brazil/Programa de Pós-Graduação em Biotecnologia Industrial, Universidade Tiradentes/Instituto de Tecnologia e Pesquisa, Aracaju, SE, 49032-490, Brazil

## Background

Actinomycetes may account for 10 to 30% of the total soil rhizosphere microorganisms. The attention given to the actinomycetes in biotechnological applications is a result of their metabolic versatility that is accompanied by the production of primary and secondary metabolites of economic importance, which are a promising source of products (*e.g.*, antibiotics, enzyme inhibitors, antiparasitic and anticancer agents) [1,2]. Included in this range of compounds are secondary metabolites synthesized by polyketide synthase (PKS) and non-ribosomal peptide synthetase (NRPS) pathways. An effective method for assessing the presence of these biosynthetic pathways is the detection of PKS and NRPS genes by PCR [3,4]. Thus, this study was based on targeted analyses of 31 soil isolate actinomycetes aiming to evaluate their antimicrobial potential through the NRPS and PKS-I pathways.

## Methods

The antimicrobial activity was evaluated by the antagonism test against two economically important phytopathogens, the bacterium *Xanthomonas campestris *and the fungus *Thielaviopsis paradoxa*, using the technique of double layer. The *X. campestris *and *T. paradoxa *were propagated at 28°C in YM (yeast malt) pH 6.0 and PDB (potato dextrose broth) respectively. The results were statistically analyzed using the Bonferroni test.

The presence of genes PKS and NRPS was evaluated by PCR, using degenerate primers for highly conserved regions encoding enzymes associated with biosynthesis of polyketides and peptides.

## Results and conclusions

According to the experimental results, 52% of the isolates showed antimicrobial activity against at least one of the target bacterial pathogens tested. Among these active isolates, some belong to rare families. Thus, this finding can be a source of novel biomolecules with antimicrobial activity. From those isolates that presented one of the NRPS and PKS-I genes, 75% of them showed antagonistic activity against one of the phytopathogens evaluated. Preliminary data on this screening demonstrate the importance of the biotechnological potential of these actinomycetes due to the antagonistic activity against plant pathogens of economic interest and the possibility of be used as biocontrol, besides offering a strong area for metabolic research [2,5].
